# Functions of the cell wall polysaccharide schizophyllan during vegetative growth of *Schizophyllum commune*

**DOI:** 10.1016/j.tcsw.2025.100167

**Published:** 2025-12-16

**Authors:** Fleur E.L. Kleijburg, Ella M. Schunselaar, Adil A. Safeer, Ajit K. Bishoyi, Marc Baldus, Han A.B. Wösten

**Affiliations:** a Microbiology, Department of Biology, Utrecht University, Padualaan 8, 3584 CH Utrecht, the Netherlands; b NMR Spectroscopy, Bijvoet Center for Biomolecular Research, Utrecht University, Padualaan 8, 3584 CH Utrecht, the Netherlands

**Keywords:** Fungus, Schizophyllum commune, Cell wall, Schizophyllan, β-(1,3)(1,6)-glucan, Environmental stress

## Abstract

Schizophyllan, a β-(1,3)(1,6)-glucan, is part of the cell wall of the mushroom-forming fungus *Schizophyllum commune* and is also released into the culture medium*.* It has various commercial applications but the natural function of schizophyllan during growth of *S. commune* is largely unknown. The *S. commune* strain H4-8A was grown on minimal medium (MM-N) and medium containing 10-fold more KH_2_PO_4_/K_2_HPO_4_ buffer (MM-NKP). The addition of extra buffer resulted in a 4.2-fold decrease in water-soluble schizophyllan and a 8.8-fold decrease in rigid schizophyllan in the cell wall. This decrease in schizophyllan was associated with a 3.7 fold lower tensile strength and a 2.5-fold higher elasticity of the mycelium. Moreover, spores of *S. commune*, as well as cells of *Escherichia coli* and *Pseudomonas putida*, showed increased survival against heat treatment and freeze-thawing, and had a longer shelf-life in the presence of schizophyllan. Also, schizophyllan can be metabolized by *S. commune* but the tested bacteria were unable to do so. Together, schizophyllan provides rigidity to the cell wall, protects *S. commune* against temperature stress, and can be used as an external carbon storage. It may also form a selective barrier around the hyphae, protecting *S. commune* against attack by bacteria.

## Highlights

• High schizophyllan production results in a stronger but less elastic cell wall.
• Schizophyllan protects *Schizophyllum commune* against temperature stress.
• Schizophyllan can be used by *Schizophyllum commune* as an external storage sugar.
• Schizophyllan may protect *Schizophyllum commune* from bacterial attack.

## 1 Introduction

The basidiomycete *Schizophyllum commune* produces a β-(1,3)(1,6)-glucan called schizophyllan (Rau et al., 1992). This exopolysaccharide consists of a β-(1,3)-glucan backbone with single β-(1,6)-glucose monomers linked to every third residue (Zhang et al., 2013) with a molecular weight up to 1.3 × 10^7^ g/mol (Rau et al., 1990). It dissolves in water as a trimer of three single chains that form a semi-flexible rod with a triple helical structure (Norisuye et al., 1980; Kashiwagi et al., 1981). This results in a highly viscous solution. Schizophyllan has a high stability, illustrated by the fact that the triple helical structure melts at temperatures >135 °C or at a pH > 12.

Schizophyllan is a major component of the fungal cell wall and forms a layer at the hyphal surface and in the space between hyphae (Kirtzel et al., 2019; Sietsma and Wessels, 1977). The cell wall of *S. commune* consists of an inner, rigid core and an outer, flexible layer (Ehren et al., 2020). Schizophyllan is a minor constituent of the inner part but a major constituent of the outer part of the *S. commune* cell wall when grown in a liquid shaken culture (Safeer et al., 2023). In these cultures, schizophyllan is also released into the medium. Production levels of schizophyllan in liquid shaking cultures have been related to oxygen limitation, inoculum size, the carbon and nitrogen source, and the pH (Kumari et al., 2008; Mohammadi et al., 2018; Rau et al., 1992; Joshi et al., 2013).

Schizophyllan has many industrial applications. It has immune-stimulatory (Sakagami et al., 1988; Zhong et al., 2015) and anti-tumor (Zhang et al., 2013) activities, which makes this polysaccharide of interest for the pharmaceutical industry (Kleijburg and Wösten, 2025). Additionally, it can be used to produce oxygen-impermeable films, for instance for food preservation (Schulz et al., 1992), as a thickener for cosmetic lotions, and as an emulsifier to enhance oil recovery (Zhang et al., 2013). Despite the commercial interest in schizophyllan, the function of this polysaccharide during vegetative growth of *S. commune* is still largely unknown. It has been shown that schizophyllan binds micro-nutrients such as Cu^2+^, Ca^2+,^ Mg^2+^, Mn^2+^, NO_3_^−^, PO_4_^3−^, and SO_4_^2−^, which may function as a storage mechanism, to reduce availability of these nutrients to competitors, and/or to prevent toxic influx in the cytoplasm (Kleijburg et al., 2023). In this study, we show that schizophyllan also provides rigidity to the cell wall, protects against temperature cycles, can act as a storage polysaccharide, and may also protect the fungal cell wall against bacterial attack.

## 2 Materials and methods

### 2.1 Fungal strain and culture conditions

The monokaryotic *S. commune* strain H4-8A (Ohm et al., 2010a) was routinely grown in the dark at 30 °C in a sealed plastic bag (20 × 30 cm; to prevent evaporation) on SCMM medium (Dons et al., 1979; Supplemental Table 1) or ammonium minimal medium (MM-N) (Kleijburg et al., 2023; Supplemental Table 1) with or without 15 g l^−1^ agar. In some experiments, MM-N was supplemented with 10-fold higher amounts of KH_2_PO_4_/K_2_HPO_4_ buffer (MM-NKP) or KCl (MM-NK) (Supplemental Table 1). To perform NMR, the glucose and (NH_4_)_2_SO_4_ in MM-N and MM-NKP were replaced with ^13^C-glucose and (^15^NH_4_)_2_SO_4_. To determine whether schizophyllan can be metabolized, the 2 % glucose in MM-N was replaced with 0.1 or 0.2 % glucose, 0.1 or 0.2 % schizophyllan, or a combination of 0.1 % glucose and 0.1 % schizophyllan, while absence of a carbon source was used as a control.

Spores of the dikaryotic *S. commune* strain H4-8AB (Ohm et al., 2010a, Ohm et al., 2010b) were used for stress experiments. To isolate these spores, H4-8AB was grown for 3 days at 30 °C on SCMM agar in the dark, after which the culture was transferred to the light and 25 °C for 4–11 days. When fruiting bodies were formed, the plate was turned up-side down overnight and spores were collected from the lid with Milli-Q water.

### 2.2 Bacterial strains and culture conditions

*Escherichia coli* K12 MG1655, *Pseudomonas putida* KT2440, *Glutamicibacter halophytocola*, *Pseudomonas helanticensis,* and *Curtobacterium flaccumfaciens* (Utrecht University collection – the latter three isolated from *Agaricus bisporus* compost) were grown at 200 rpm and 30 °C (*P. putida)* or 37 °C (other species) in LB medium (10 g l^−1^ tryptone powder, 5 g l^−1^ yeast extract, 10 g l^−1^ NaCl). To assess growth on schizophyllan, the bacteria were grown overnight in 30 ml tubes containing 5 ml SV medium (7 mg l^−1^ (NH4)2FeSO4. 6H2O, 0.05 g l^−1^ MgSO4.7H2O, 2 g l^−1^ NH4Cl, 4.38 g l^−1^ KH2PO4, 7.81 g l^−1^ K2H2PO4, 0.2 % glucose). Cultures were 100-fold diluted in 200 μl SV medium without carbon source, 0.1 or 0.2 % glucose, 0.1 or 0.2 % schizophyllan, or a combination of 0.1 % glucose and 0.1 % schizophyllan and grown in flat-bottom 96-wells plates for 24 h at 150 rpm. OD_600_ was measured with the Biotek Synergy HTX spectrophotometer (Agilent Technologies, www.agilent.com).

Soil samples collected in Utrecht (GPS coordinates 52.085170 5.169813 and 52.096492 5.133314) were suspended in Milli-Q water (0.1 g ml^−1^) and filtered using Whatmann paper. The flow-through was diluted 100-fold in SV medium without carbon source, or with 0.2 % glucose or schizophyllan and grown for 12 days in flat-bottom 96-wells plates. The OD_600_ was measured as described above. Ten-day-old cultures grown in 0.1 % schizophyllan were diluted 100- and 1000-fold and grown for 7 days after spreading 100 μl on SV plates with 0.1 % schizophyllan as a carbon source.

### 2.3 Schizophyllan production

MM-N, MM-NK and MM-NKP agar plate cultures were inoculated with a plug from the periphery of a 7-day old MM-N agar colony placed directly on the agar or on a 76 mm polycarbonate track etched (PCTE) membrane (0.1 μm pore size) (GvS, www.gvs.com) overlaying the medium. After growing for 7 days, the mycelium was washed overnight at 20 rpm on a C1 Platform Shaker (New Brunswick Scientific, www.nbsc.com) followed by centrifugation for 10 min at 10,000 g. The mycelium in the pellet was washed with water twice for 1 min and harvested by centrifugation at 3200 *g* for 5 min after each wash. Schizophyllan in the supernatant of the overnight wash was precipitated by addition of ethanol to a final concentration of 30 % *v*/v, vigorous shaking for 5 s, and overnight incubation at 4 °C. This was followed by centrifugation for 10 min at 10,000 g, after which the polysaccharide in the pellet was freeze-dried and weighed.

### 2.4 Purification of schizophyllan

Mycelium (12 cm^2^ of a 7-day-old SCMM agar culture) was macerated in 50 ml SCMM for 30 s at 18000 rpm in a Waring 2 Speed Blender. The macerated mycelium was incubated for 24 h at 200 rpm and macerated again. A total of 0.2 g wet weight macerated mycelium was used to inoculate 100 ml SCMM in 250 ml Erlenmeyers. Cultures were grown for 7 days at 200 rpm, after which the mycelium was pelleted for 30 min at 4 °C and 10,000 g and washed three times with 40 ml dH_2_O, each wash followed by centrifugation. The supernatant from each centrifugation cycle was pooled and filtered using Miracloth. Schizophyllan in the pooled fractions was precipitated by adding 30 % ethanol (see above), pelleted by centrifugation, and washed twice with 30 % ethanol (each wash followed by centrifugation at 10,000 *g*). Residual ethanol was evaporated at 60 °C for 8 h and the polysaccharide was dialyzed twice for 2 h and once overnight against 50 volumes dH_2_O at 4 °C in a 12 kDa cellulose dialysis membrane (Merck, www.sigmaaldrich.com). Purity of the isolated schizophyllan was confirmed using Fourier-Transform Infrared (FTIR) spectroscopy and ^1^H-detected solid state NMR (ssNMR) (Supplementary Fig. 1). For FTIR spectroscopy, 2 mg freeze-dried schizophyllan mixed with 250 mg KBr was dried overnight at 60 °C and analyzed in a VerTex 70 FTIR Spectrometer (Bruker, www.bruker.com). FTIR absorbance peaks at ∼3400, ∼2900, ∼1650 and ∼ 1100 cm^−1^ and peaks ranging from ∼1500 to ∼1200 cm^−1^ (Supplemental Fig. 1) validated the purity of the schizophyllan (Mohammadi et al., 2018). For ^1^H-detected ssNMR, schizophyllan (6 mg ml^−1^) was lyophilized, stored overnight in a desiccator, and then packed into 1.3 mm Magic Angle Spinning rotors. The ^1^H-detected dipolar hCH experiment was conducted on a narrow bore 700 MHz (16.4 T) spectrometer at 60 kHz MAS at Tset of 260 K using a 1.3 mm HXY MAS probe (Bruker Biospin). Due to frictional heating, the actual sample temperature was calibrated to 293 K using a KBr powder sample (Thurber and Tycko, 2009). PISSARO decoupling (Weingarth et al., 2008) was applied for the dipolar experiment, while the MISSISSIPI pulse sequence (Zhou and Rienstra, 2008) was used for water suppression. Acquisition parameters for the experiments are provided in Supplemental Fig. 1. Spectral processing was conducted using Bruker Topspin 3.6.2 software. Chemical shifts for ^1^H and ^13^C were referenced to the water resonance and adamantine, respectively, and analyzed using the NMRFAM-Sparky software (Lee et al., 2015). Only signals were found for β-(1,3)/(1,6) glucan, confirming the purity of the polysaccharide.

### 2.5 The effect of schizophyllan on freeze-thawing and heat treatment of microbial cells

*S. commune* spores and *E. coli* and *P. putida* cells (grown overnight in 20 ml LB in 100 ml Erlenmeyers) were washed three times in 20 ml Milli-Q water with centrifugation at 4000 *g* for 5 min in between each wash. Spores or cells were suspended in 1 ml Milli-Q water (10^4^ ml^−1^) without schizophyllan or with 0.02 or 0.05 % schizophyllan and subjected to three types of temperature cycles; cycles between 4 °C and 25 °C, cycles between room temperature and 45 °C (*S. commune*), 50 °C (*E. coli*) or 40 °C (*P. putida*) and cycles of freezing and thawing. The first 4 °C - 25 °C cycle consisted of an overnight incubation at 4 °C followed by a 1.5 h incubation at 25 °C, while the second and third cycle consisted of an incubation of 1.5 h at 4 °C followed by an incubation for 1.5 h at 25 °C. The three 20 °C - 45 °C (*S. commune*), 50 °C (*E. coli*) or 40 °C (*P. putida*) cycles consisted of incubations for 30 min at high temperature followed by 30 min at 20 °C. The first freeze-thaw cycle was done overnight at −20 °C and 1.5 h at 25 °C; the second and third cycle were done with 1.5 h incubations at −20 °C and 25 °C each. The colony forming units (CFU) were determined after each temperature cycle. The treated *S. commune* spores (100 μl of each sample) were grown on SCMM agar for 3 days, while the treated bacteria (5 and 50 μl of each sample) were grown overnight on LB agar.

### 2.6 Mechanical analysis of the mycelium

A total of 1 g wet weight macerated mycelium (see above) was taken up in 3 ml MM-N or MM-NKP and spread on a 76 mm PCTE membrane on top of MM-N or MM-NKP agar, respectively. Cultures (6 for each condition) were grown for 12 days and the resulting mycelium was stacked on top of each other on the lid of a Petri dish and dried for 5 days. The first 5 colonies were placed with their air-exposed side down (facing the Petri dish), while the last colony was placed with this side facing up.

Tensile measurements were conducted on dog bone shapes cut from the dried mycelium with an ISO 527 type 5A sample cutter in a ZCP 020 manual cutting press (Zwick, www.zwick-gmbh.de). The thickness of the sample was measured at the ends and middle of the dog bone shapes with a digital Metro MT 1200 length gauge device (Heidenhain, www.heidenhain.com). The weight of the sample was divided by the volume (area of the shape × average thickness) to determine the density of the sample. Tensile strength was measured at room temperature by clamping the samples 10 mm from each end in a Zwick/Roell ZO20 (Zwick GmbH, www.zwick-gmbh.de) with a 1 kN load cell, a pre-load force of 0.25 N, and a 2 mm min^−1^ test speed. The Young's modulus was calculated in the linear part of the stress/strain curve, the tensile strength was calculated by dividing the maximum load at break by the area (thickness × width in mm^2^) of the sample, and elongation was calculated from the elongation of the sample (mm) at break.

### 2.7 Cell wall composition determined by NMR

To determine the cell wall composition of *S. commune* on various media, H4-8A was grown on agar or liquid medium with ^13^C-glucose and ^15^NH_4_. For agar cultures, an agar plug taken from the periphery of a 7-day old MM-N agar colony was grown for 7 days on a PCTE membrane placed on top of MM-N or MM-NKP agar with ^13^C-glucose and ^15^NH_4_. For liquid shaken cultures, 0.1 g wet weight macerated mycelium (see above) was grown for 7 days at 200 rpm in 50 ml MM-N with ^13^C-glucose and ^15^NH_4_ in a 250 ml Erlenmeyer. The mycelium of agar cultures was removed from the PCTE membrane, while the mycelium of the liquid shaken cultures was harvested by centrifugation for 10 min at 10,000 g. The mycelia were freeze-dried and homogenized in a SK550 1.1 heavy-duty paint shaker (Fast & Fluid, www.fast-fluid.com) for 9 min in a 15 ml (agar culture) or 50 ml (liquid culture) Greiner tube with 5 (agar culture) or 10 (liquid culture) 4.76 mm (85 mg) metal beads (Bofix, www.bofix.nl). The cell walls were washed four times with 10 (agar culture) or 40 (liquid culture) ml dH_2_O with centrifugation at 10,000 *g* for 10 min between the washes.

Fast magic angle spinning (MAS) solid-state NMR (ssNMR) was used to study the composition of cell walls (Kleijburg et al., 2023; Safeer et al., 2023) using a narrow bore 16.4 T (700 MHz) NMR spectrometer with a 1.3 mm HXY MAS probe (Bruker BioSpin, see section 2.4). Temperatures were set at 258 K at 54 kHz MAS frequencies, which resulted in an actual sample temperature of 298 K due to frictional heating (Thurber and Tycko, 2009). For dipolar-based ^1^H—^13^C 2D experiments that probed rigid cell wall components a ^13^C offset of 51 ppm was used with a spectral width of 130 ppm. A 70–100 % forward and backward cross-polarization (CP) ramps were used with 1.2 ms and 0.2 ms CP contact times, respectively. For the dipolar-based experiments, PISARRO (Weingarth et al., 2009) decoupling was applied at 15 kHz. For scalar-based ^1^H—^13^C 2D experiments that sampled flexible cell wall signals from polysaccharides and amino acid side chains a ^13^C offset of 57.3 ppm was used with a spectral width of 130 ppm, while WALTZ16 (Shaka et al., 1983) decoupling was performed at 10 kHz. For all experiments 90° pulses were applied in the 147–178 kHz range for ^1^H and in the 89–91 kHz range for ^13^C and max. 200 ms of water suppression was achieved using MISSISSIPPI at 23 kHz (Zhou and Rienstra, 2008). ^1^H and ^13^C chemical shifts were referenced to the water peak at 4.7 ppm and to an external adamantane reference, respectively.

TopSpin 4.1 (Bruker BioSpin) was used to process and analyze NMR data. Peaks were integrated to determine the relative abundance of rigid cell wall species in dipolar-based 2D ^1^H—^13^C spectra. Errors were based on the signal-to-noise of integrated peaks. For the relative abundance analysis of flexible polymer species in scalar-based 2D ^1^H—^13^C spectra, cumulative peak intensities per cell wall polymer were considered (Safeer et al., 2023), and flexible polysaccharide abundance was determined using only the representative polysaccharide peaks in the carbohydrate C1 region (^13^C dimension, 90–110 ppm range). Errors were based on the signal-to-noise of peak intensities.

### 2.8 Interaction of calcofluor white-stained schizophyllan with the cell surface of *S. commune* spores and bacterial cells

An aqueous solution of schizophyllan (6 mg ml^−1^) was mixed with an equal volume of Calcofluor White Stain (Sigma-Aldrich, www.sigma-aldrich.com) and incubated for 5 min at room temperature in the dark. The suspension was dialyzed 3 times for 1 h against 100 volumes dH_2_0. The Calcofluor White-stained schizophyllan (20 μl) was mixed with 4 × 10^6^ spores or bacteria (20 μl) that had been washed 3 times with 1 ml Milli-Q water and taken up in 8.5 g l^−1^ NaCl. The mixtures were incubated for 10 min at room temperature in the dark. Interaction of the schizophyllan with the spores and bacterial cells was visualized with a Zeiss Axioscope 5 microscope (ZEISS, www.zeiss.com) with the UV light set to 50 % and an exposure time of 10 ms.

### 2.9 Statistics

Two-sample *t*-tests (in case of equal variance) or Welch two-sample t-tests (in case of unequal variance) were used to analyze all experiments (*p* ≤ 0.05). In all cases, biological triplicates were used.

## 3 Results

### 3.1 pH of the culture medium impacts schizophyllan production

*S. commune* is typically grown on SCMM medium using asparagine as nitrogen source (Dons et al., 1979). This medium does not allow effective ^15^N labelling for ssNMR studies and, therefore, MM-N medium was used, in which asparagine is substituted with (^15^NH_4_)_2_SO_4_ (Safeer et al., 2022; Kleijburg et al., 2023; Supplemental Table 1). However, this substitution impacts the pH of the medium during culturing. Normally, when *S. commune* H4-8A is grown on SCMM agar, the pH of the culture medium drops from 7 to 5 (our unpublished data), but it drops from 6.8 to 2.9 on MM-N (Table 1). To prevent this drop in pH, H4-8A was also grown on MM-NKP medium, which contains a 10-fold higher amount of KH_2_PO_4_/K_2_HPO_4_ buffer. When growing on MM-NKP, the pH dropped from 7.2 to 6.4 (Table 1). Growing on MM-NKP also resulted in a 4.2-fold reduction (g^−1^ mycelium) in the production of water-soluble schizophyllan when compared to MM-N (Table 1). This reduction in schizophyllan production could be the result of the pH of the medium or the addition of extra K^+^ and PO_4_^3−^. To distinguish between these effects, H4-8A was grown on MM-NKP agar of which the starting pH was reduced to 6.4 (MM-NKP low pH) or on MM-N agar that was supplemented with 10-fold KCl with a pH of 6.4 (MM-NK). The pH dropped to 5.2 and 3.1 during culturing, respectively, while the production of schizophyllan resembled that on MM-N agar (Table 1). Together, these results show that more schizophyllan is produced when the pH of the culture medium becomes ≤5.2.

**Table 1:** Production of biomass and schizophyllan, and pH of the culture medium, and lifting phenotype (i.e. the possibility to lift the mycelium from the agar medium) of 7-day-old *S. commune* cultures grown on MM-N, MM-NKP, MM-NKP low pH or MM-NK agar. Averages are indicated with SEM, while letters indicate statistical significance between treatments (p ≤ 0.05).

	Mycelium (g)	Schizopyllan (mg g^−1^ biomass)	pH	Lifting phenotype
MM-NKP	0.04 ± 0.00 (a)	9.10 ± 1.72 (a)	6.4 ± 0.0 (a)	Yes
MM-N	0.09 ± 0.01 (b)	37.78 ± 3.99 (b)	2.9 ± 0.0 (b)	No
MM-NKP low pH	0.08 ± 0.01 (b)	27.58 ± 6.12 (b)	5.2 ± 0.2 (c)	Yes
MM-NK	0.05 ± 0.00 (a)	33.18 ± 1.69 (b)	3.1 ± 0.0 (d)	No

**Fig. 1:**
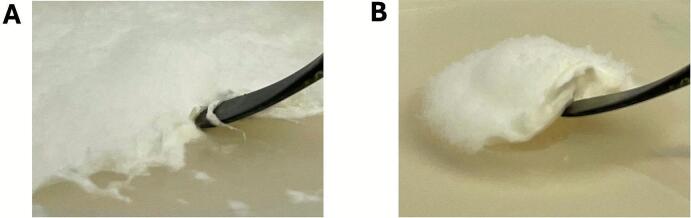
A 7-day culture of H4-8A cannot be lifted from MM-N agar (A) but is easily lifted in the case of MM-NKP agar (B).

Besides the difference in schizophyllan production on various media types, we also observed a difference in the possibility to remove the mycelium from the agar medium. Mycelium of a 7-day-old H4-8A colony grown directly on MM-N or MM-NK agar could not be removed from the medium with a spatula (Fig. 1A). By contrast, the mycelium could be easily lifted from the medium after growing on MM-NKP or MM-NKP low pH. Together, there is no relation between the amount of schizophyllan produced on agar medium and the ability to adhere to this medium.

### 3.2 Cell wall composition of *S. commune* on MM-N and MM-NKP

**Fig. 2:**
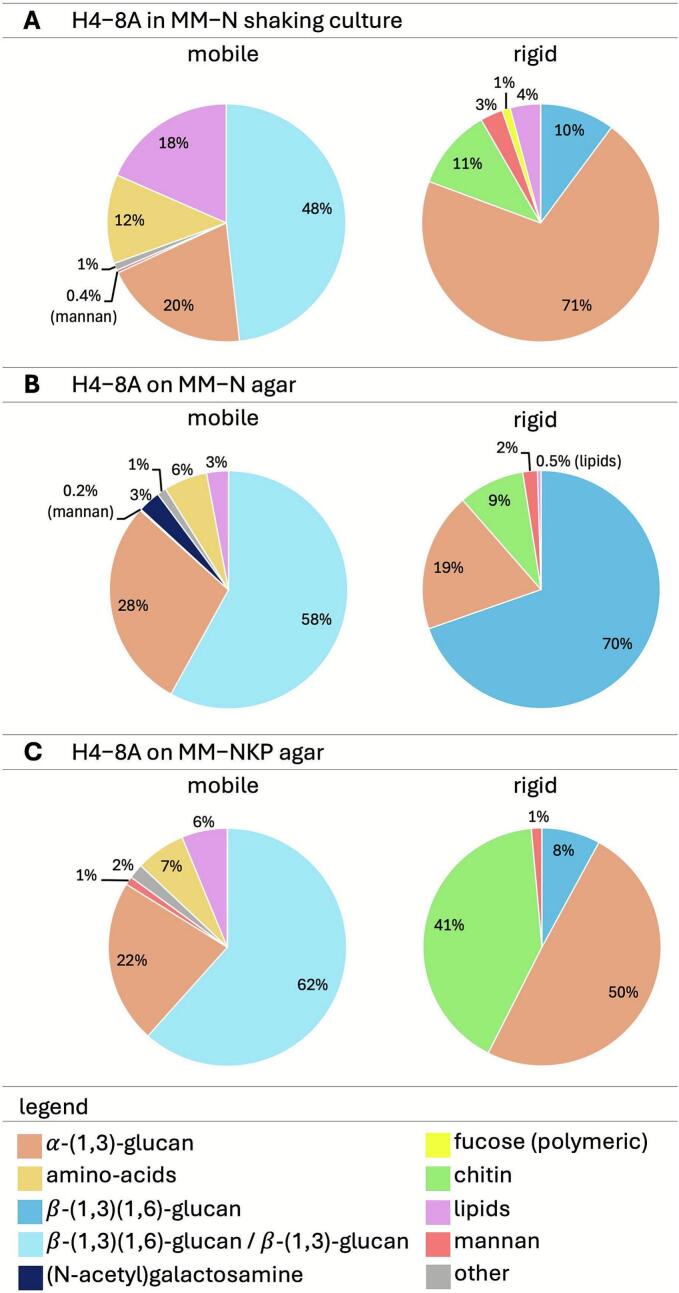
Quantitative assessment of the rigid and mobile cell wall of H4-8A grown in a liquid shaken MM-N culture or on MM-N and MM-NKP agar media.

Strain H4-8A was grown on ^13^C-glucose, ^15^NH_4_ MM-N and MM-NKP agar and in a ^13^C-glucose, ^15^NH_4_ MM-N liquid shaking culture. Composition of the mobile and rigid fractions of the water-washed cell walls of these cultures were assessed by ssNMR (Fig. 2; Supplemental Fig. 2). The composition of the mobile and rigid parts of the cell walls of the MM-N liquid shaken culture were in good agreement with that of a liquid shaken culture published previously (Safeer et al., 2023). The main components of the outer, mobile part of the cell wall of the liquid shaken MM-N culture consisted of β-(1,3)/(1,6)-glucan and α-(1,3)-glucan with a contribution of 48 % and 20 %, respectively (Fig. 2; Supplemental Fig. 2), while these values were 59 % and 31 %, respectively, in the study of Safeer et al. (2023). The main components of the inner, rigid fraction of the cell wall of the liquid shaken culture consisted of α-(1,3)-glucan, chitin, β-(1,3)(1,6)-glucan (schizophyllan) and mannan with a contribution of 71 %, 11 %, 10 %, and 3 %, respectively (Fig. 2; Supplemental Fig. 2), and 57 %, 8 %, 7 %, and 5 % (Safeer et al., 2023). It should be noted that β-(1,3)(1,6)-glucan and β-(1,3)-glucan cannot be distinguished in scalar-based ^1^H—^13^C 2D experiments that probe the mobile cell wall components and are therefore here collectively called β-(1,3)/(1,6)-glucan. Lipids made up 4 % and 9 % (Safeer et al., 2023) and 18 % and 4 % (Fig. 2; Supplemental Fig. 2) of the mobile and rigid parts of the cell wall, respectively. These compounds probably originate from the plasma membrane (Safeer et al., 2023) and will therefore not be further discussed.

Like cell walls of MM-N liquid shaken cultures, β-(1,3)/(1,6)-glucan and α-(1,3)-glucan were the most dominant polymers in the outer, mobile cell wall of MM-N and MM-NKP agar cultures (Fig. 2). This part of the cell wall consisted of 58 % and 62 % β-(1,3)/(1,6)-glucan, respectively, and 20 % and 28 % α-(1,3)-glucan. The only difference observed in this part of the cell wall was the presence of 3 % (*N*-acetyl)galactosamine in the case of the MM-N agar culture, while this sugar was not observed in the other two conditions. Like cell walls of MM-N liquid shaken cultures, α-(1,3)-glucan, chitin, β-(1,3)(1,6)-glucan and mannan made up the inner, rigid cell wall of MM-N and MM-NKP agar cultures. However, there was more variation in the amounts of these components in these two medium conditions. The rigid cell wall fraction of mycelium grown on MM-N agar contained 70 % β-(1,3)(1,6)-glucan, 19 % α-(1,3)-glucan, 9 % chitin, and 2 % mannan. By contrast, the amount of β-(1,3)(1,6)-glucan on MM-NKP agar was only 8 %, while α-(1,3)-glucan and chitin were higher with 50 % and 41 % and mannan was similar with 1 %. Together, addition of 10-fold KH_2_PO_4_/K_2_HPO_4_ reduces the amount of schizophyllan in the rigid cell wall, while it increases the amount of α-(1,3)-glucan and chitin.

### 3.3 Mechanical analysis of the mycelium

Density of mycelium grown on MM-N (324 kg m^−3^) and MM-NKP (336 kg m^−3^) agar were similar (Table 2). Mechanical analysis was performed to determine the Young's modulus, tensile strength and elongation of break of these mycelia (Table 2; Supplemental Fig. 3). There was a trend (*p* = 0.09) showing a higher Youngs modulus for mycelium grown on MM-N agar (235 MPa) compared to MM-NKP agar (24 MPa). Additionally, tensile strength was 3.7-fold higher and elongation at break was 2.5-fold lower for mycelium grown on MM-N (1.51 MPa and 0.94 %) compared to MM-NKP agar (0.40 MPa and 2.32 %). Together, growth on MM-NKP medium decreases the tensile strength but increases the elongation at break of the mycelium.

**Table 2:** Density, Young’ s modulus, tensile strength, and elongation at break of 12-day-old H4-8A mycelium grown on MM-N or MM-NKP agar. Averages are indicated with SEM, while letters indicate significance between treatments (p ≤ 0.05).

	Density (kg m^3^)	Young's modulus (MPa)	Tensile strength (MPa)	Elongation at break (%)
MM-N	324 ± 14(a)	235 ± 69 (a)	1.51 ± 0.16 (a)	0.94 ± 0.19 (a)
MM-NKP	336 ± 14 (a)	24 ± 6 (a)	0.40 ± 0.09 (a)	2.32 ± 0.0.29 (b)

### 3.4 Effect of schizophyllan on the growth of *S. commune* and bacteria

**Fig. 3:**
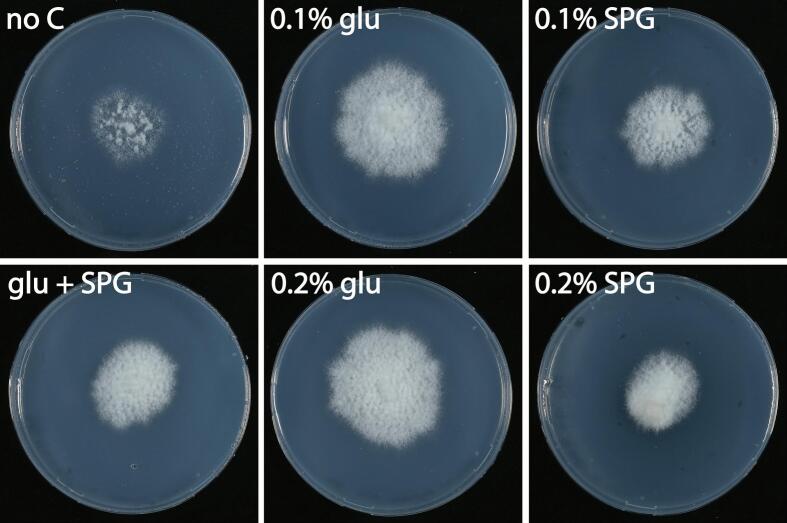
Seven-day-old colonies of *S. commune* grown on MM-N agarose without carbon source (no C), with 0.1 % or 0.2 % glucose (0.1 %, 0.2 % glu), 0.1 % or 0.2 % schizophyllan (0.1 % or 0.2 % SPG), or a combination of 0.1 % glucose with 0.1 % schizophyllan (0.1 % glu + 0.1 % SPG).

To determine whether *S. commune* can utilize schizophyllan as a carbon source, *S. commune* H4-8A was grown on MM-N agarose with 0.1 % or 0.2 % glucose or schizophyllan, or a combination of 0.1 % glucose and 0.1 % schizophyllan. *S. commune* showed some growth on the medium without carbon source, while growth on glucose was highest. Yet, clear growth was also observed on 0.1 % and 0.2 % schizophyllan and on 0.1 % glucose +0.1 % schizophyllan (Fig. 3).

**Fig. 4:**
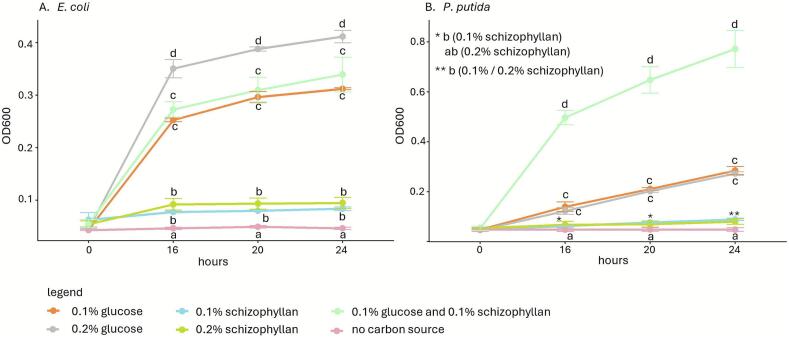
Growth of *E. coli* (A) and *P. putida* (B) in SV without carbon source or with glucose and/or schizophyllan. Averages are indicated with SEM, while letters indicate significance between treatments (p ≤ 0.05).

To determine whether bacteria can also utilize schizophyllan as a carbon source, *E. coli*, *P. putida G. halophytocola*, *P. helanticensis,* and *C. flaccumfaciens* were grown on SV with no carbon source, with 0.1 % or 0.2 % glucose, 0.1 % or 0.2 % schizophyllan, or a combination of 0.1 % glucose and 0.1 % schizophyllan. Minimal (*E. coli* and *P. putida*) to no (*G. halophytocola*, *P. helanticensis* and *C. flaccumfaciens*) growth was observed in SV medium with no carbon source or schizophyllan as sole carbon source (Fig. 4, Supplemental Fig. 4). Growth of *G. halophytocola*, *P. helanticensis,* and *C. flaccumfaciens* on a combination of 0.1 % glucose and 0.1 % schizophyllan was in all cases 2- to 3-fold lower than on 0.1 % or 0.2 % glucose. Growth of *E. coli* was similar on 0.1 % glucose and 0.1 % glucose with 0.1 % schizophyllan, while growth of *P. putida* was 2.8 to 3.5-fold higher on 0.1 % glucose with 0.1 % schizophyllan compared to 0.1 % glucose.

**Fig. 5:**
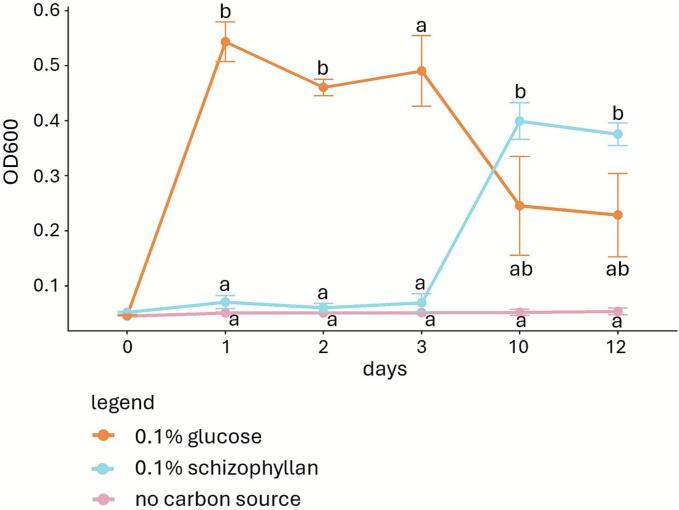
Growth of 100-fold dilutions of soil filtrate during 12 days in SV with no carbon source, glucose, or schizophyllan. Averages are indicated with SEM, while letters indicate significance between treatments (p ≤ 0.05).

To isolate microbes capable of metabolizing schizophyllan, we conducted a selective enrichment of soil samples using schizophyllan as a carbon source. Diluted and filtered soil samples were incubated in SV medium with no carbon source, with 0.1 % glucose or with 0.1 % schizophyllan. On medium without carbon source, no growth was observed after 12 days (Fig. 5). With 0.1 % glucose, the OD_600_ increased up to 0.5 after 24 h, while with 0.1 % schizophyllan the OD_600_ reached 0.4 after 10 days (Fig. 5). The culture grown in SV with 0.1 % schizophyllan was serially diluted and plated on SV agar with 0.1 % schizophyllan as carbon source. Microscopic analysis revealed protozoa-like organisms growing on the plate rather than bacterial colonies. Indeed, there is evidence that some protozoa can degrade β-(1–3)-glucan (Rosengaus et al., 2014; Nishimura et al., 2020). Together, these results indicate that *S. commune* can metabolize schizophyllan, but that the bacteria used in this study can hardly, if at all, use this polysaccharide as a carbon source.

### 3.5 Schizophyllan promotes survival of *S. commune* spores and bacteria during temperature cycles

**Fig. 6:**
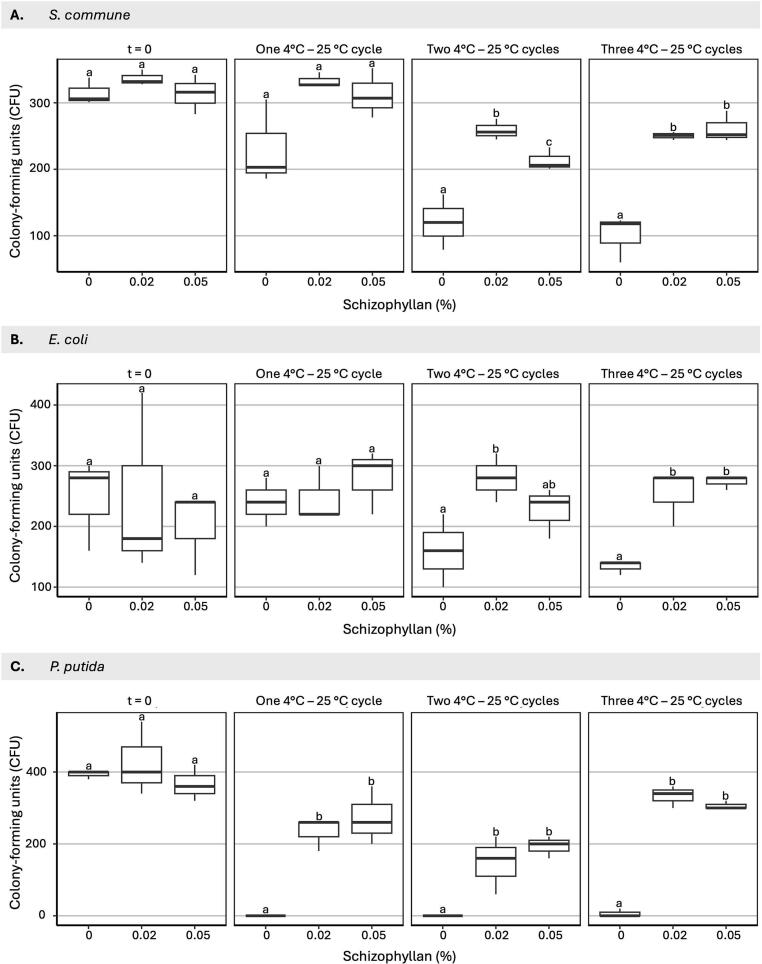
Schizophyllan improves the shelf-life at 4 °C of *S. commune* spores and *E. coli* or *P. putida* cells. A total of 10^4^ H4-8A *S. commune* spores (A), *E. coli* cells (B), or *P. putida* cells (C) were diluted in 1 ml water or in 0.02 and 0.05 % schizophyllan and exposed to 1–3 4 °C–25 °C cycles. The first cycle consisted of an overnight incubation at 4 °C followed by a 1.5 h incubation at 25 °C, while the second and third cycle consisted of an incubation of 1.5 h at 4 °C followed by an incubation for 1.5 h at 25 °C. For *S. commune* the same starting cultures were used for Figs. 6, 7 and 8. For *E. coli* and *P. putida* the same starting cultures were used for Figs. 6 and 8. Averages are indicated with SEM, while different letters indicate significance between CFUs and schizophyllan concentrations at each temperature cycle (p ≤ 0.05)

We examined the effect of addition of schizophyllan on survival of *S. commune* and bacterial cells after exposure to various temperature regimes. Because quantification of CFUs of mycelial fragments is difficult, we used *S. commune* spores to more easily quantify survival of the fungus. First, the fungal spores and bacterial cells were exposed to cycles of 4 °C and 25 °C in the absence or presence of 0.02 or 0.05 % schizophyllan. These amounts of schizophyllan are in the range found in the culture medium. After two and three cycles, survival of *S. commune* spores was ∼2-fold higher in schizophyllan compared to water (Fig. 6). Survival of *E. coli* was >2-fold higher in schizophyllan after three cycles, while survival of *P. putida* was even ≥44-fold higher in schizophyllan than in water after one, two and three cycles.

**Fig. 7:**
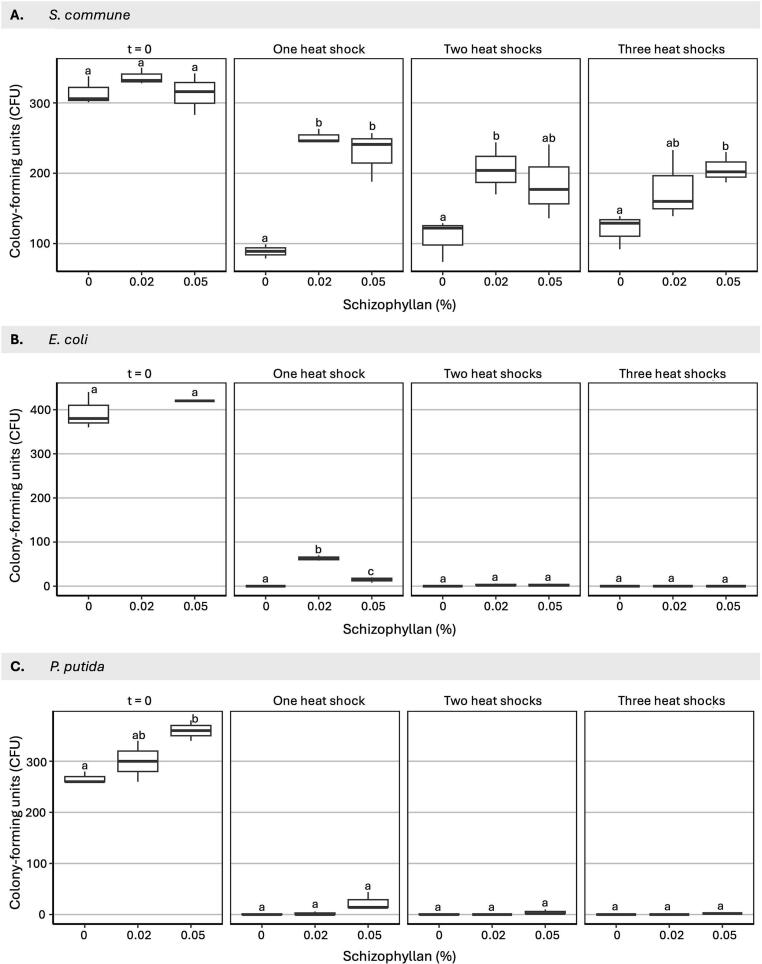
Schizophyllan protects *S. commune* spores, and *E. coli* and *P. putida* cells against heat stress. A total of 10^4^ *S. commune* H4-8A spores (A), *E. coli* cells (B), or *P. putida* cells (C) were taken up in 1 ml water or 0.02 and 0.05 % schizophyllan and exposed to 1, 2, and 3 heat treatments. Each heat treatment consisted of a 30 min exposure at 40 °C for *P. putida* cells, 45 °C for *S. commune* spores, or 50 °C for the *E. coli* cells, followed by a 30 min incubation at room temperature. For *S. commune* the same starting cultures were used for Figs. 6, 7 and 8. For *E. coli t* = 0 in 0.02 % schizophyllan colony-forming units could not be counted due to biofilm formation. Averages are indicated with SEM, while different letters indicate significance between CFUs and schizophyllan concentrations at each temperature cycle (p ≤ 0.05).

Next, *S. commune* spores, and *P. putida* and *E. coli* cells were exposed to a heat treatment at 45, 40, and 50 °C, respectively. These temperatures killed ≥75 % of the cells in the first heat-treatment cycle in the absence of schizophyllan. After one, two, or three heat exposures, survival of *S. commune* spores in 0.02 or 0.05 % schizophyllan was 1.7 to 2.7-fold higher than in water (Fig. 7). In water, *E. coli* did not survive heat exposure at all, while 63 and 15 *E. coli* cells (15.5 % and 3.6 %) survived one heat exposure in 0.02 or 0.05 % schizophyllan, respectively. However, survival was low (< 1 %) and even absent after two and three heat treatments, respectively. Only <0.3 % *P. putida* cells survived one or two heat treatments in water, while all cells were killed after three heat treatments. Addition of 0.02 % schizophyllan did not improve survival but 7.8 % of the *P. putida* cells survived one heat treatment in 0.05 % schizophyllan, while survival after two and three heat treatments was not significantly different from the control.

**Fig. 8:**
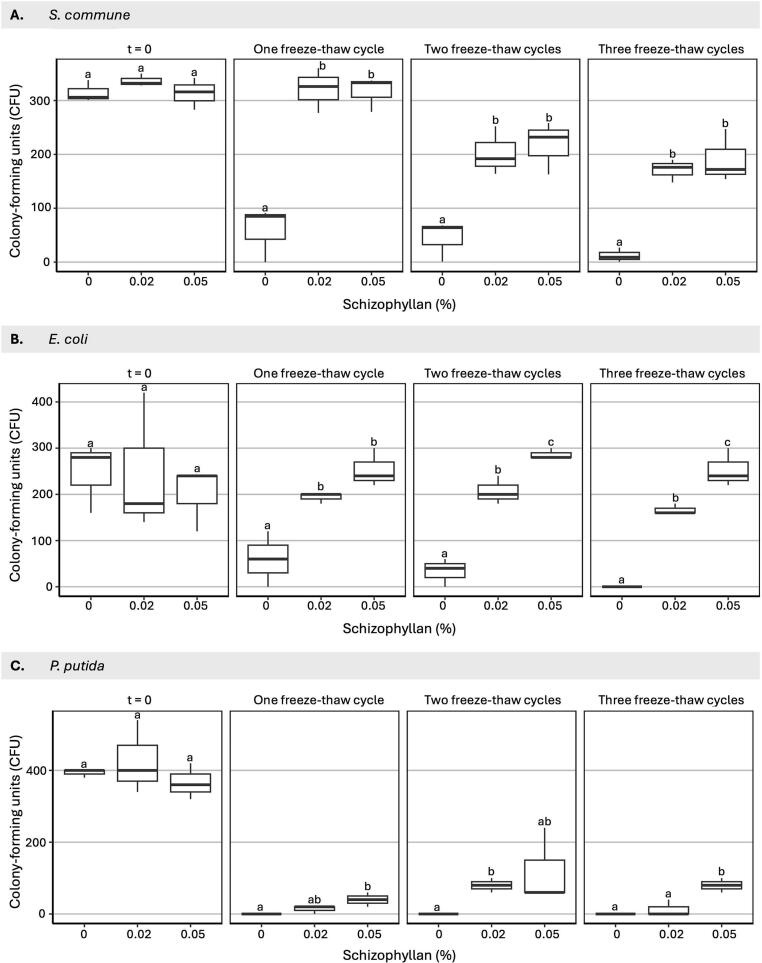
Schizophyllan protects *S. commune* spores and *E. coli* or *P. putida* cells against freeze-thaw cycles. A total of 10^4^ *S. commune* H4-8A spores (A), *E. coli* cells (B), or *P. putida* cells (C) were taken up in 1 ml water or in 0.02 and 0.05 % schizophyllan and exposed to 1, 2, and 3 freeze-thaw cycles. The first cycle consisted of an overnight incubation at −20 °C followed by a 1.5 h incubation at 25 °C, while the second and third cycle consisted of an incubation of 1.5 h at −20 °C followed by an incubation for 1.5 h at 25 °C. For *S. commune* the same starting cultures were used for Figs. 6, 7 and 8. For *E. coli* and *P. putida* the same starting cultures were used for Figs. 6 and 8. Averages are indicated with SEM, while different letters indicate significance between CFUs and schizophyllan concentrations at each temperature cycle (p ≤ 0.05).

Finally, *S. commune* spores and bacterial cells were exposed to 1–3 freeze-thaw cycles in the absence or presence of 0.02 or 0.05 % schizophyllan. After one, two, or three freeze-thaw cycles, survival of *S. commune* spores and *E. coli* cells in schizophyllan compared to water was 3.2-fold to >15.5-fold higher (Fig. 8). For *P. putida*, no cells survived in water, while 3.0–18.7 % and 10.9–32.7 % of the cells survived in the presence of 0.02 % and 0.05 % schizophyllan, respectively, after one to three freeze-thaw cycles.

*S. commune* spores and *E. coli* and *P. putida* cells were incubated in 0.02 or 0.05 % schizophyllan stained with Calcofluor White. The spores became fluorescent but the bacterial cells did not, showing that schizophyllan is not forming a protective envelope in the case of these bacteria (Supplemental Fig. 5). Notably, temperature increase of 0.02 % or 0.05 % schizophyllan was delayed when compared to pure water when these solutions were incubated in a water bath at 50 °C (Supplemental Fig. 5). Together, results indicate that schizophyllan can promote the survival of microbial cells during temperature cycles by a reduced temperature transfer in the viscous polysaccharide solution.

## 4 Discussion

Schizophyllan is present in both the inner, rigid part and the outer, mobile part of the cell wall (Ehren et al., 2020; Safeer et al., 2023) and forms a layer at the hyphal surface and in the space between hyphae (Kirtzel et al., 2019; Sietsma and Wessels, 1977). It has been shown that schizophyllan binds micro-nutrients, which may function as a storage mechanism, to reduce availability of these nutrients to competitors, and/or to prevent toxic influx in the cytoplasm (Kleijburg et al., 2023). In this study, it is shown that schizophyllan also provides ductility to the cell wall, protects against temperature cycles, and can act as a storage polysaccharide. Furthermore, it may protect the fungal cell wall against bacterial attack.

Growth of *S. commune* on MM-NKP (i.e. MM-N with a 10-fold amount of KH_2_PO_4_/K_2_HPO_4_ buffer) resulted in a moderate drop in pH (from 7.2 to 6.4) during culturing. This was accompanied by a 4.2-fold reduction (g^−1^ mycelium) in the production of water-soluble schizophyllan when compared to MM-N. In the latter medium, the pH dropped from 6.8 to 2.9. By growing *S. commune* on MM-NKP low pH (that has a lower starting pH than MM-NKP) and on MM-NK (that has a concentration of potassium similar to MM-NKP) it was shown that schizophyllan production is increased when the pH of the medium decreases to ≤5.2 during culturing. During these growth experiments it was noticed that *S. commune* grown on MM-N or MM-NK agar could not be removed from the medium with a spatula, while this was the case when the fungus was grown on MM-NKP or MM-NKP low pH. The absence of a relation between the production of water-soluble schizophyllan and the adherence of the mycelium to the agar medium implies that this polysaccharide is not involved in adherence to this substrate. Possibly, the absence of polymers containing (*N*-acetyl)galactosamine in the outer, mobile part of the cell wall of MM-NKP cultures (see below) is responsible for the lack of adherence to the agar medium. This hypothesis is based on results in *Aspergillus fumigatus.* The cell wall of hypo-adherent mutant strains of this fungus contain less *N*-acetylgalactosamine relative to the wild type strain (Gravelat et al., 2013). It has also been shown that deacetylation of a polysaccharide of *N*-acetylgalactosamine and galactose (called galactosaminegalactan) in the cell wall of *A. fumigatus* enables adhesion of the mycelium to surfaces (Bamford et al., 2020).

The reduced production of schizophyllan in MM-NKP was also evident in the composition of the rigid fraction of the cell wall of *S. commune*. Solid state NMR showed that α-(1,3)-glucan, chitin, and schizophyllan were the main polymers in this inner part of the cell wall after growth on MM-N or MM-NKP agar. However, the amount of schizophyllan was 8.8-fold lower in the latter medium, while the amount of α-(1,3)-glucan and chitin was 2.6-fold and 4.6-fold higher, respectively. Notably, the composition of the mobile cell wall fraction of *S. commune* grown on MM-N or MM-NKP agar was similar. This outer part of the cell wall consisted of 58–62 % schizophyllan / β-(1,3)-glucan and 20–28 % α-(1,3)-glucan. The only difference observed in this part of the cell wall was the presence of 3 % (*N*-acetyl)galactosamine in the case of the MM-N agar culture, while this sugar was not observed in the MM-NKP agar culture. The difference in cell wall composition probably impacted the mechanical properties of the mycelium. A reduced amount of schizophyllan in the inner, rigid part of the cell wall MM-NKP correlated with a higher ductility and lower tensile strength of the mycelium. Therefore, schizophyllan in the inner part of the cell wall seems to have a structural function, and thereby is expected to contribute to cell shape and mechanical protection of hyphae.

We wondered whether schizophyllan also has a functional role in the outer part of the cell wall or when released into the medium. *Phoma herbarum* isolated from Antarctica produces an extracellular glucan with β-1,3 and β-1,6 linkages, which protects the fungus from inactivation due to repeated freezing and thawing (Selbmann et al., 2002). In the present study, we assessed whether extracellular schizophyllan has a similar role. It was shown that schizophyllan protects *S. commune* spores against freeze-thaw cycles and heat shock(s). In fact, it even increased spore survival during less severe temperature changes, with cycles between 4 and 25 °C. Interestingly, schizophyllan also protected *E. coli* and *P. putida* against temperature stress. Incubation of *S. commune* spores and *E. coli* and *P. putida* cells in 0.02 % or 0.05 % schizophyllan labelled with Calcofluor White revealed a fluorescent envelop around the fungal spores but not around the bacterial cells. Therefore, the latter cells were not protected from temperature stress by a schizophyllan envelope. Notably, schizophyllan (0.02 % or 0.05 %) delayed temperature increase when water was incubated at 50 °C in a water bath, which is explained by a reduced convection in viscous solutions (Ramamurthy and Krishnan, 2022). This delayed temperature increase may provide temperature stress protection. Schizophyllan may also reduce/prevent ice crystal formation during freezing, thereby protecting cells. A range of polysaccharides have been shown to reduce/prevent ice-crystal formation (Sun et al., 2022).

*S. commune* was shown to be able to grow on schizophyllan and this polysaccharide may therefore serve as an extracellular storage sugar. Extracellular storage of a reserve sugar has the risk that other microbes benefit as well. However, the bacteria that were tested in this study hardly, if at all, grew on schizophyllan. Indeed, only few bacteria that produce β-(1,3)-glucanases have been reported (Ferrer, 2006; Labourel et al., 2014; Lim et al., 1991; Yang et al., 2020). On the other hand, other microorganisms like protozoa may still feed on the schizophyllan (Rosengaus et al., 2014; Nishimura et al., 2020). The inability of (a large) part of the bacteria to grow on schizophyllan may provide protection of *S. commune*. Bacteria that cannot grow on this polysaccharide at the hyphal surface will have reduced or totally inhibited growth, thereby preventing lysis of the fungal cell wall by bacterial enzymes such as chitinase.

## CRediT authorship contribution statement

**Fleur E.L. Kleijburg:** Writing – review & editing, Writing – original draft, Methodology, Investigation, Formal analysis, Conceptualization. **Ella M. Schunselaar:** Writing – review & editing, Writing – original draft, Methodology, Investigation, Formal analysis, Conceptualization. **Adil A. Safeer:** Writing – original draft, Methodology, Investigation, Formal analysis, Conceptualization. **Ajit K. Bishoyi:** Writing – review & editing, Investigation, Formal analysis. **Marc Baldus:** Writing – review & editing, Supervision, Funding acquisition, Conceptualization. **Han A.B. Wösten:** Writing – review & editing, Supervision, Funding acquisition, Conceptualization.

## Funding

This work was funded by the Dutch Research Council NWO domain Applied and Engineering Sciences (grant number 18425), the Fungateria HORIZON-EIC-2021-PATHFINDER CHALLENGES project (Grant agreement 101071145) as well as the National Roadmap Large-Scale NMR Facility of the Netherlands (uNMR-NL grid, grant number 184.035.002).

## Declaration of competing interest

The authors declare that they have no known competing financial interests or personal relationships that could have appeared to influence the work reported in this paper.

## Data Availability

Raw data can be found at https://doi.org/10.5281/zenodo.17078287

## Appendix A Supplementary data

Supplementary material: Supplemental Table 1 and Supplemental figures 1-5. Image 1
