# Supplementary material for: Functions of the cell wall polysaccharide schizophyllan during vegetative growth of Schizophyllum commune
Source: Cell Surf. 2025 Dec 16;15:100167. doi: 10.1016/j.tcsw.2025.100167 (PMC12771330; doi:10.1016/j.tcsw.2025.100167)
Supplement: Supplementary file 1 — Supplementary material: Supplemental Table 1 and Supplemental figures 1-5. [file mmc1.docx]

**Supplemental data**

**Supplemental Table 1.** Composition of the *S. commune* media used in the study.

| **Nutrient** | **MM-N (mg l^-1^)** | **MM-NKP (mg l^-1^)** | **MM-NK (mg l^-1^)** | **(SCMM) (mg l^-1^)** |
| --- | --- | --- | --- | --- |
| BO_3_^3-^ | 0.06 | 0.06 | 0.06 | 0.06 |
| Ca^2+^ | 0.20 | 0.20 | 0.20 | 0.20 |
| Cl^-^ | 3.40 | 3.40 | 33.98 | 3.40 |
| Co^2+^ | 0.10 | 0.10 | 0.10 | 0.10 |
| Cu^2+^ | 0.05 | 0.05 | 0.05 | 0.05 |
| Fe^3+^ | 1.72 | 1.72 | 1.72 | 1.72 |
| K^+^ | 586.26 | 586.26 | 5862.64 | 586.26 |
| Mg^2+^ | 49.30 | 49.30 | 49.30 | 49.30 |
| Mn^2+^ | 0.02 | 0.02 | 0.02 | 0.02 |
| MoO_4_^2-^ | 0.05 | 0.05 | 0.05 | 0.05 |
| NH_4_^+^ | 360.42 | 360.42 | 360.42 | NA |
| NO^3-^ | 0.63 | 0.63 | 0.63 | 0.63 |
| PO_4_^3-^ | 872.54 | 8725.42 | 872.54 | 872.54 |
| SO_4_^2-^ | 1155.24 | 1155.24 | 1155.24 | 195.66 |
| Zn^2+^ | 0.45 | 0.45 | 0.45 | 0.45 |
| glucose | 20000 | 20000 | 20000 | 20000 |
| thiamine | 0.12 | 0.12 | 0.12 | 0.12 |
| L-asparagine | NA | NA | NA | 1319.97 |

**
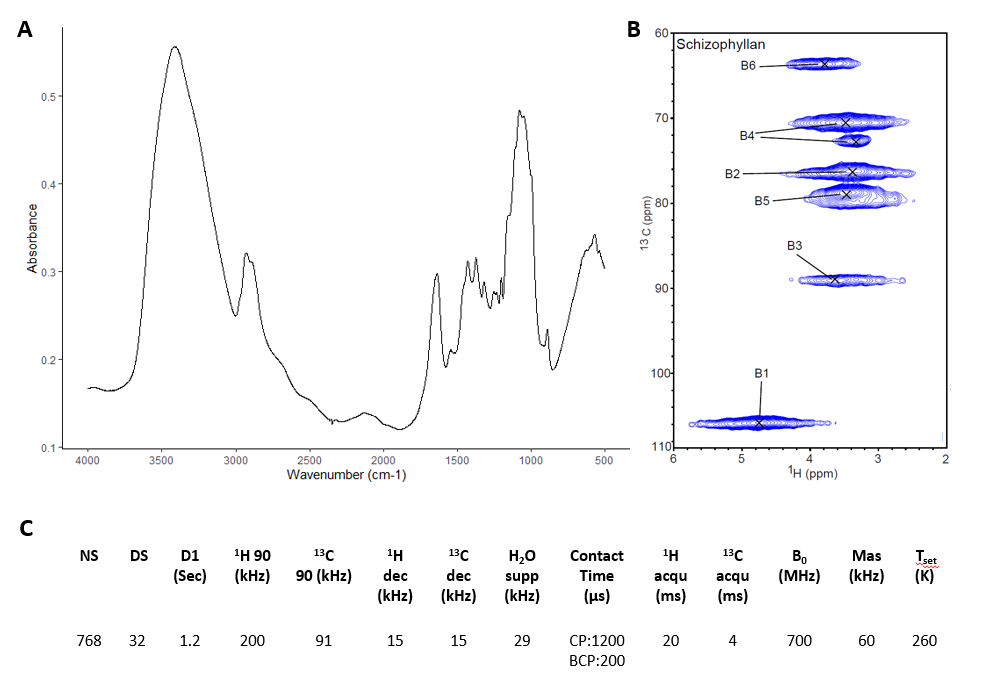
**

**Supplemental Figure 1.** Fourier transformed infrared spectroscopy (A) and ¹H-detected dipolar hCH solid-state NMR (B) experiments of purified schizophyllan. (C) Acquisition parameters of the solid-state NMR experiment (NS: number of scans, DS: Number of Dummy scans, D1: recycle delay, ^1^H90 and ^13^C90: R.F. strength of ^1^H and ^13^C 90 degree pulses, respectively. ^1^H. ^13^CDec: Decoupling field strength of ^1^H and ^13^C pulses. H_2_O: R.F. strength of H_2_O water suppression pulse. Contact times for CP and BCP cross polarization, ^1^H and ^13^C acquisition times in milliseconds. B_0_ field strength, MAS rate in kHz and sample temperature in K). Peak assignments in B1-B6 in (B) depict the proton and carbon signals of β-(1,3)/(1,6)-glucan. Peak assignments in B1-B6 (B) depict the proton and carbon signals of β-(1,3)/(1,6)-glucan.


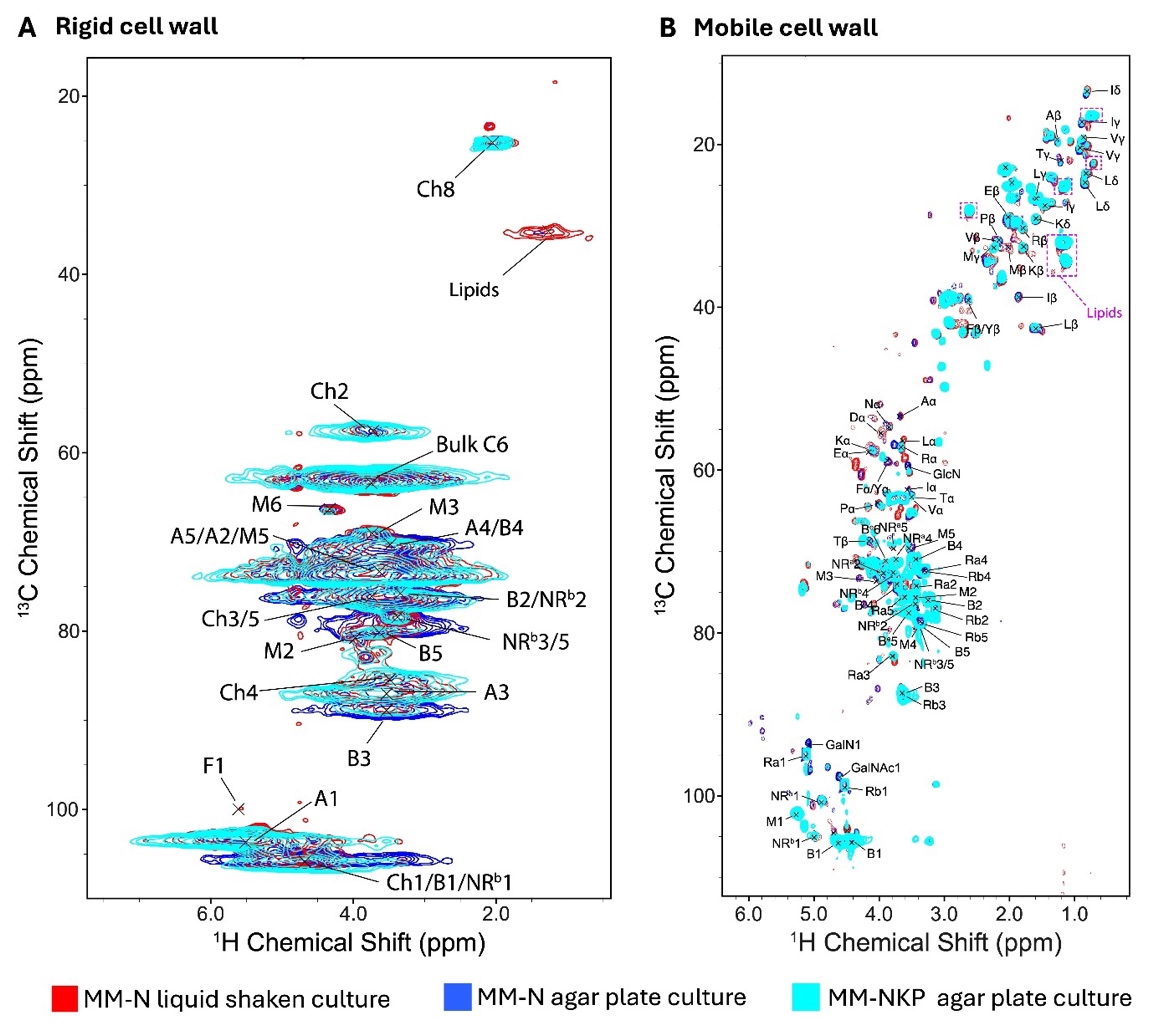
**Supplemental Figure 2.** ssNMR spectra of the rigid (A) and mobile (B) cell wall of H4-8A grown in liquid shaken MM-N culture (red), on MM-N agar (red) or on MM-NKP agar media (cyan).


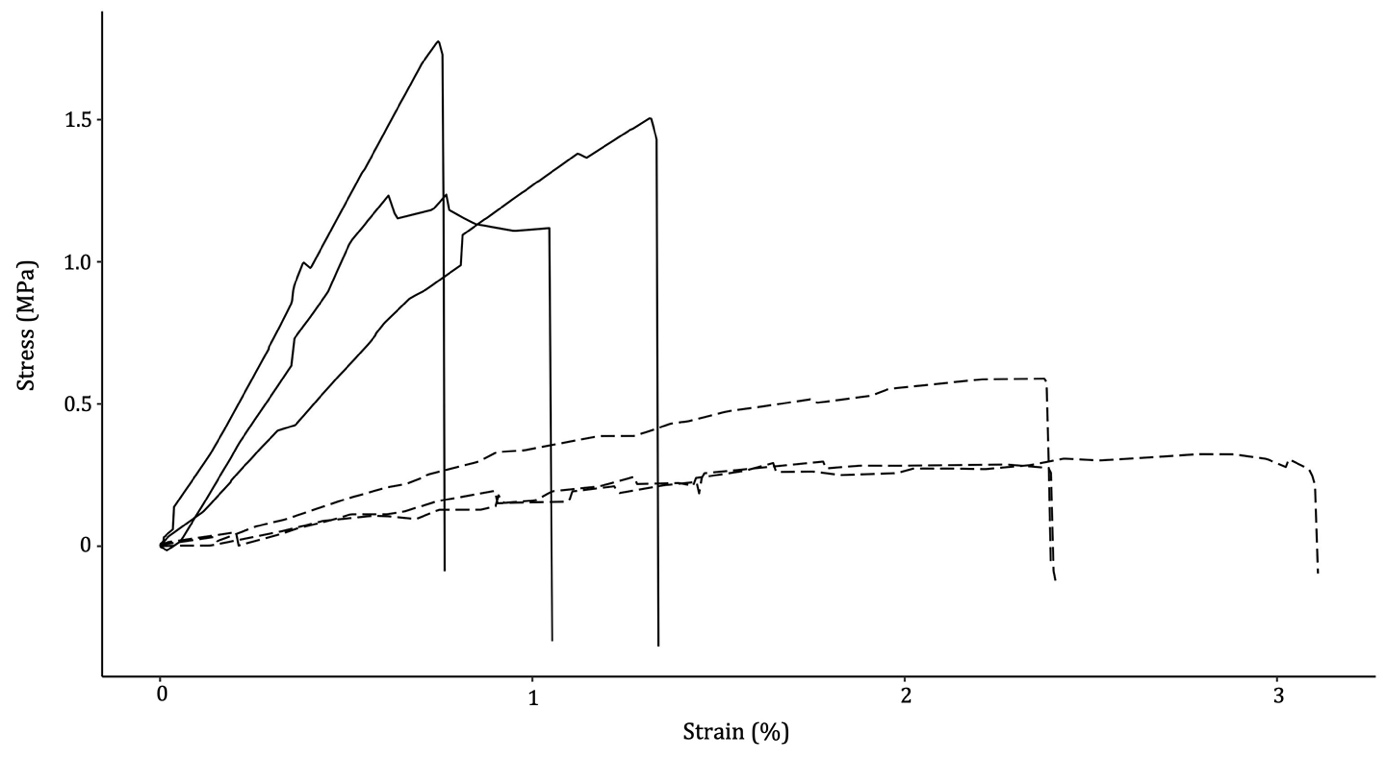


**Supplemental Figure 3**. Stress strain curves of 12-day-old H4-8A mycelium sheets grown on MM-N (solid lines) and MM-NKP (dashed lines) agar.


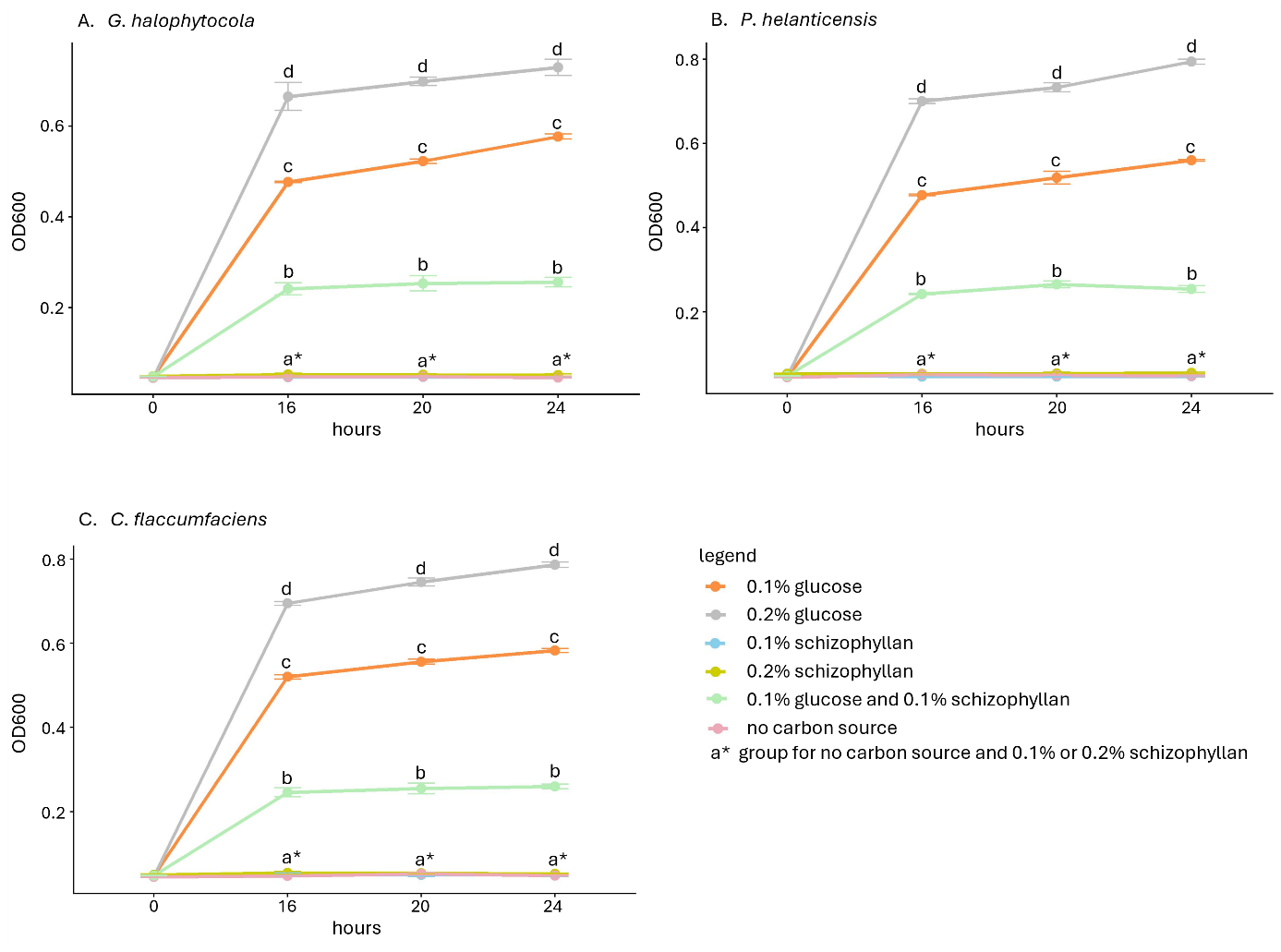


**Supplemental Figure 4**. Growth of *G. halophytocola* (A), *P. helanticensis* (B) and *C. flaccumfaciens* (C) in SV without carbon source (grey) or with glucose and / or schizophyllan (blue, brown and green). Averages are indicated with SEM, while letters indicate significance at each time point (p $\leq$ 0.05).


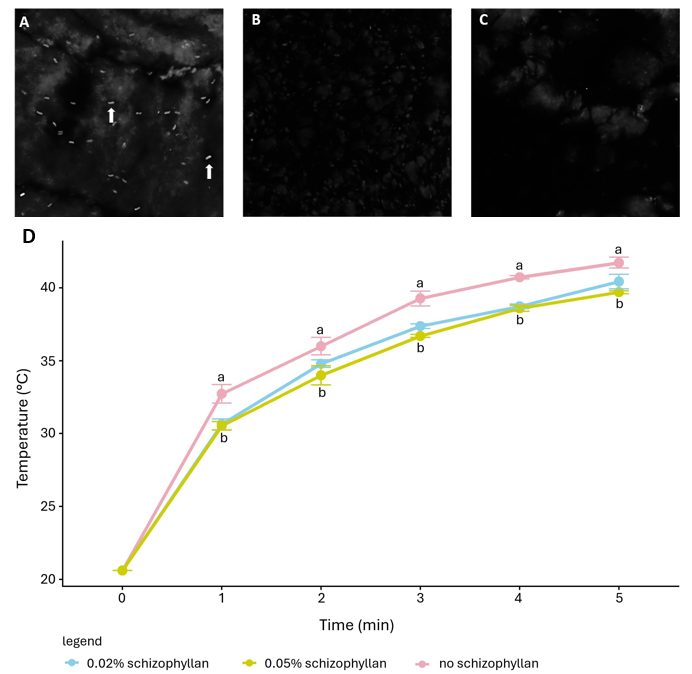


**Supplemental Figure 5.** Mechanism of heat protection by schizophyllan. Spores of *S. commune* (A) and cells of *E. coli* (B) and *P. putida* (C) were incubated with Calcofluor White-stained schizophyllan. The spores were stained with a layer of schizophyllan but the bacterial cells were not. (D) Increase in temperature of water, 0.02% schizophyllan, and 0.05% schizophyllan when incubated in a water bath at 50 °C. Water warmed up more quickly than the schizophyllan containing solutions (≤ 0.05). Averages are indicated with SEM, while letters indicate significance at each time point (p $\leq$ 0.05).
